# Feasibility of a Mobile Phone App and Telephone Coaching Survivorship Care Planning Program Among Spanish-Speaking Breast Cancer Survivors

**DOI:** 10.2196/13543

**Published:** 2019-07-09

**Authors:** Anna María Nápoles, Jasmine Santoyo-Olsson, Liliana Chacón, Anita L Stewart, Niharika Dixit, Carmen Ortiz

**Affiliations:** 1 Division of Intramural Research National Institute on Minority Health and Health Disparities National Institutes of Health Bethesda, MD United States; 2 Division of General Internal Medicine Department of Medicine University of California San Francisco San Francisco, CA United States; 3 Center for Aging in Diverse Communities Institute for Health and Aging University of California San Francisco San Francisco, CA United States; 4 Division of Hematology/Oncology Department of Medicine University of California San Francisco/Zuckerberg San Francisco General Hospital San Francisco, CA United States; 5 Círculo de Vida Cancer Support and Resource Center San Francisco, CA United States

**Keywords:** Hispanic Americans, cancer survivors, mobile apps, feasibility studies

## Abstract

**Background:**

Spanish-speaking Latina breast cancer survivors experience disparities in knowledge of breast cancer survivorship care, psychosocial health, lifestyle risk factors, and symptoms compared with their white counterparts. Survivorship care planning programs (SCPPs) could help these women receive optimal follow-up care and manage their condition.

**Objective:**

This study aimed to evaluate the feasibility, acceptability, and preliminary efficacy of a culturally and linguistically suitable SCPP called the Nuevo Amanecer (New Dawn) Survivorship Care Planning Program for Spanish-speaking breast cancer patients in public hospital settings, approaching the end of active treatment.

**Methods:**

The 2-month intervention was delivered via a written bilingual survivorship care plan and booklet, Spanish-language mobile phone app with integrated activity tracker, and telephone coaching. This single-arm feasibility study used mixed methods to evaluate the intervention. Acceptability and feasibility were examined via tracking of implementation processes, debriefing interviews, and postintervention satisfaction surveys. Preliminary efficacy was assessed via baseline and 2-month interviews using structured surveys and pre- and postintervention average daily steps count based on activity tracker data. Primary outcomes were self-reported fatigue, health distress, knowledge of cancer survivorship care, and self-efficacy for managing cancer follow-up health care and self-care. Secondary outcomes were emotional well-being, depressive and somatic symptoms, and average daily steps.

**Results:**

All women (n=23) were foreign-born with limited English proficiency; 13 (57%) had an elementary school education or less, 16 (70%) were of Mexican origin, and all had public health insurance. Coaching calls lasted on average 15 min each (SD 3.4). A total of 19 of 23 participants (83%) completed all 5 coaching calls. The majority (n=17; 81%) rated the overall quality of the app as “very good” or “excellent” (all rated it as at least “good”). Women checked their daily steps graph on the app between 4.2 to 5.9 times per week. Compared with baseline, postintervention fatigue (B=–.26; *P=*.02; Cohen *d*=0.4) and health distress levels (B=–.36; *P=*.01; Cohen *d*=0.3) were significantly lower and knowledge of recommended follow-up care and resources (B=.41; *P=*.03; Cohen *d*=0.5) and emotional well-being improved significantly (B=1.42; *P=*.02; Cohen *d*=0.3); self-efficacy for managing cancer follow-up care did not change. Average daily steps increased significantly from 6157 to 7469 (B=1311.8; *P=*.02; Cohen *d*=0.5).

**Conclusions:**

We found preliminary evidence of program feasibility, acceptability, and efficacy, with significant 2-month improvements in fatigue, health distress, and emotional well-being and increased knowledge of recommended follow-up care and average daily steps. Tailored mobile phone and health coaching SCPPs could help to ensure equitable access to these services and improve symptoms and physical activity levels among Spanish-speaking Latina breast cancer survivors.

## Introduction

### Background

Women with breast cancer are living longer, and the number of survivors is increasing as the US population ages. Recognizing the need to address the long-term needs of cancer survivors, in 2006, the Institute of Medicine recommended that all cancer patients receive a survivorship care plan (SCP) with a summary of their treatments, follow-up care plan, and information on potential late effects, self-care, and resources [1]. In 2016, the American College of Surgeons Committee on Cancer developed an accreditation standard requiring cancer care programs to provide SCPs to all nonmetastatic patients treated with curative intent with annual evaluation of these plans [2]. However, providing patients with SCPs is ineffective unless cancer patients understand and know how to use this information.

Survivorship care planning programs (SCPPs), to be distinguished from SCPs alone, are patient-centered activation interventions providing information on recommended health care and self-care following cancer treatment [1]. SCPPs typically help patients understand and follow recommended care regimens and encourage healthy lifestyles. SCPPs can meet patients’ information needs [3], improve communication with clinicians, and improve well-being [4]. In addition, SCPPs need to address healthy lifestyles as most cancer survivors tend to be overweight or obese and have sedentary lifestyles [5-7], particularly Latinos, [8] and strong observational evidence links these risk factors with poorer survival among breast cancer survivors [9]. Physical activity interventions, in particular, improve symptoms and health-related quality of life [10-12] and reduce the risk of recurrence and death among breast cancer survivors [13]. However, clinicians rarely provide lifestyle counseling to cancer survivors despite evidence that oncologists’ recommendations are effective among cancer survivors [14,15].

Non-white cancer survivors, in particular, face ongoing informational needs to address fear of recurrence and management of symptoms, late effects of treatments, and lifestyle changes [16]. Latina breast cancer survivors experience disparities in knowledge of breast cancer survivorship, psychosocial health, lifestyle risk factors, and symptoms after treatment compared with their white counterparts [17-21]. Spanish-speaking Latina breast cancer survivors, especially, report many unmet medical, psychosocial, and informational needs that affect negatively their self-efficacy for managing survivorship [22-24]. SCPPs could help these women receive optimal care and manage their condition. Preliminary evidence suggests high acceptability of mobile health (mHealth) apps among Latino cancer patients because of a high need for Spanish-language information and support on disease and treatment effects [25].

### Objectives

The objectives of this mixed-methods study were to develop and evaluate the feasibility, acceptability, and preliminary efficacy of a culturally and linguistically suitable SCPP called the Nuevo Amanecer (New Dawn) Survivorship Care Planning Program for Spanish-speaking breast cancer patients in public hospital settings as they approach the end of active treatment. The intervention was delivered via a written SCP and booklet, mobile phone app, and telephone coaching calls and aimed to decrease fatigue and health distress and increase knowledge and self-efficacy for managing cancer survivorship and physical activity levels.

## Methods

We describe the intervention components and then methods for examining feasibility, acceptability, and preliminary efficacy.

### Intervention

The 2-month intervention comprised 4 components: (1) hard copy of an individualized bilingual SCP, (2) Spanish-language survivorship information booklet, (3) Spanish-language mobile app called trackC with integrated activity tracker (Fitbit Zip), and (4) 5 weekly health coaching telephone calls in Spanish to reinforce survivorship care concepts and positive health behaviors. Combined, these components were designed to provide a support system for women’s cancer survivorship needs. On the basis of Social Cognitive Theory, the individually tailored intervention was designed to improve outcomes by building self-efficacy for managing cancer (managing stress and fatigue by walking, recognizing symptoms, securing follow-up services, and communicating with physicians), using self-regulation tools of self-monitoring, goal setting, and feedback [26].

#### Written Spanish Language Survivorship Care Plan

We adapted the American Clinical Society of Oncology (ASCO) SCP template [27] for low-literacy, Spanish-speaking Latinas, simplifying the layout and translating it into Spanish. Adaptations were based on iterative review by a Latina psycho-oncologist, 2 oncologists, a bilingual oncology nurse, and 3 Spanish-speaking breast cancer survivors. Participants signed a medical release form, and study personnel extracted the information from medical records to complete the SCP. Completed SCPs were reviewed by the project director and the patient’s oncologist or oncology nurse and scanned into the patient’s electronic health record. This written bilingual SCP was given to participants at the second home visit.

#### Spanish-Language Survivorship Information Booklet

We selected the “ASCO Answers: Cancer Survivorship” guide because it was comprehensive, easy to understand, and available in English and Spanish [28]. The guide covers what to expect after active treatment, including psychological, physical, sexual, reproductive, financial, and work-related challenges.

#### TrackC Mobile App With Integrated Activity Tracker

**Figure 1:**
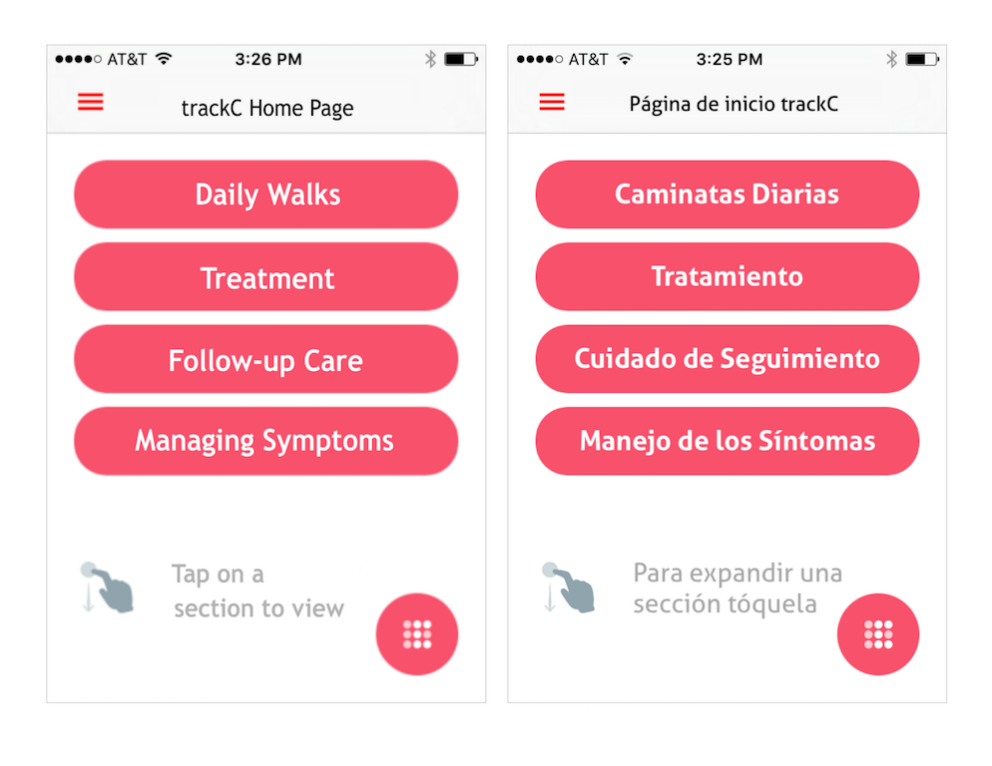
trackC mobile app home page: Caminatas Diarias (Daily Walks), Tratamiento (Treatment), Cuidado de Seguimiento (Follow-up Care), and Manejo de los Síntomas (Managing Symptoms).

**Figure 2:**
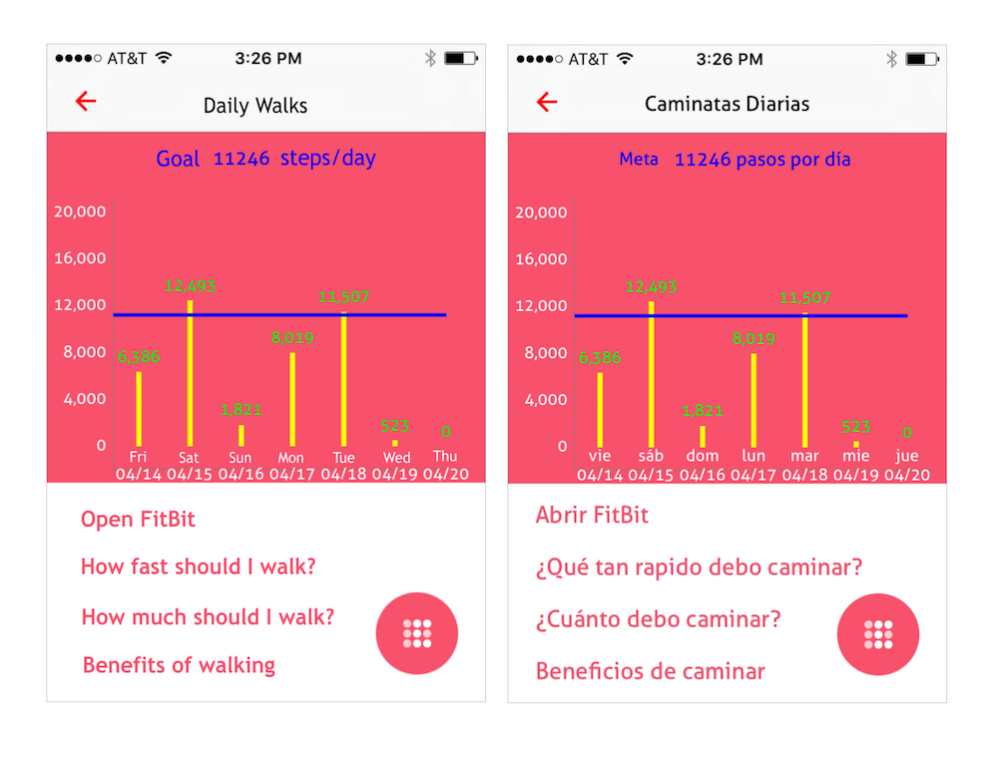
Average daily steps graph of the trackC mobile app.

The Spanish-language mobile app (trackC) was designed to contain women’s breast cancer diagnostic and treatment history and provide information on potential side effects, healthy lifestyles, and survivorship resources. An activity tracker was integrated with the app to display progress toward a personalized daily steps goal. We selected the Fitbit Zip wireless activity tracker, henceforth referred to as activity tracker, based on cost, simplicity, and availability of an application programming interface (API, for integrating the Fitbit with other software applications). The mobile app home page contained 4 section tabs (Figure 1): Daily walks (caminatas diarias), treatment (tratamiento), follow-up care (cuidado de seguimiento), and managing symptoms (manejo de los síntomas). Content was based on ASCO treatment guidelines at the time. We summarize each section briefly:

- Daily walks: information on walking and integrated activity tracker that could be synced with the app so that it displayed a history of daily steps and their average daily steps target (Figure 2).
- Treatment: screens for entering cancer diagnosis and treatment information that could be updated as needed and emailed to others, including clinicians.
- Follow-up care: general follow-up recommendations for women with noninvasive breast cancer; specific follow-up recommendations for those receiving radiation, tamoxifen, aromatase inhibitors, and women experiencing premature menopause; option to record pending medical appointments and receive reminder notifications.
- Managing symptoms: information on signs of recurrence, treatment side effects, daily exercise, nutrition, and cancer survivorship resources.

#### Developing and Testing TrackC

In phases, we developed mock-ups, a detailed wire frame, and a prototype of the app employing user-centered testing [29] with iterative review and pretesting by 3 Spanish-speaking Latina breast cancer survivors; a Latina psycho-oncologist (breast cancer survivor); an oncologist serving ethnically diverse, low-income cancer patients; and 6 bilingual-bicultural study staff members. The prototype was developed in English and then translated into Spanish using rigorous forward translation and team reconciliation methods.

#### Health Coaching Protocol

The coaching protocol was based on evidence-based motivational interviewing and health coaching techniques, which seek to actively engage patients in managing their health within their social contexts [30]. The health coach encouraged use of trackC, walking, reporting symptoms to clinicians, and calling clinicians to ask questions. Communication with clinicians was emphasized because of evidence that Latino patients often lack the confidence to report symptoms or ask questions, especially when the physician speaks a different language [31,32]. The health coach reinforced cancer survivorship information. The health coach was a bilingual-bicultural Latin American–trained internist with extensive health coaching experience. Coaching consisted of 5 weekly phone calls with the following structure: (1) review of progress toward daily steps goal and working through any barriers, (2) daily steps goal setting for the coming week, and (3) information on a weekly health topic. The 5 health topics paralleled the trackC content and included: (1) walking and nutrition, (2) breast cancer follow-up care, (3) signs of recurrence, (4) treatment side effects, and (5) resources and review of content from the first 4 calls. The health coach used a manual, but tailored the content based on participants’ needs.

### Study Design and Procedures

This single-arm feasibility study was conducted between February and June 2017, with women recruited from 2 public hospitals in Northern California. All study materials, including the app, were translated into Spanish using team translation and expert review and reconciliation by 6 bilingual-bicultural research staff. The study provided all participants with an iPhone and covered the costs of the data plan. Participants were compensated a total of $60 for completing 2 assessments (baseline and post intervention). During the 2-month study, the same trained bilingual-bicultural research associate (RA) conducted 3 scheduled home visits: (1) enrollment visit (baseline assessment), (2) 1-week visit at the end of the activity tracker run-in period, and (3) final end-of-study visit (postintervention assessment). This protocol was approved by the University of California San Francisco and Contra Costa Regional Medical Center and Health Centers institutional review boards.

#### Eligibility and Recruitment

Eligibility criteria consisted of: (1) self-reported Spanish-speaking Latina, (2) diagnosed with nonmetastatic breast cancer, and (3) within 1 year of termination of active treatment (except for hormonal therapy). Exclusion criteria included walking more than 30 min on 5 days per week or more. Using lists of potentially eligible participants provided by the hospital sites, we mailed them bilingual initial contact letters and postage-paid refusal postcards. A total of 2 weeks later, women who had not returned a refusal postcard were contacted in person or on the telephone by trained bilingual-bicultural RAs to conduct eligibility screening, ask about mobile phone usage, and schedule an appointment to visit the participant’s home within 1 week.

#### Study Enrollment—Home Visit 1

The RA conducted the enrollment visit (45-60 min) at the clinic site or the participant’s home during which the study was explained in detail, written informed consent was obtained, participants signed a medical release form, and the baseline survey was completed. This marked the start of the 1-week run-in period. Women were provided with a masked activity tracker (hidden daily steps display) and instructions to wear it every day for a minimum of 10 hours per day and not to change their usual activity levels. This run-in period was used to establish participants’ baseline average daily steps and personalized goal (average daily steps during run-in period + 2000 steps).

#### End of 1 Week Run-In Period—Home Visit 2

In this 1-hour visit, participants received materials and verbal instructions on the use of the written SCP; survivorship booklet; iPhone and charger with trackC app installed; unmasked activity tracker (with visible daily steps and goal graph); and a step-by-step illustrated guide on how to use the iPhone, app, and activity tracker devices. The RA reviewed the SCP, survivorship booklet, device guide, and the individualized average daily steps goal to be achieved within 2 months. Women were instructed on synchronizing the tracker and mobile app at the end of every day to update the app’s average daily steps graph. The RA helped participants enter diagnostic and treatment information from the written SCP into trackC.

#### End of Study—Home Visit 3

At this visit, the RA conducted the final assessment and a brief satisfaction survey, synchronized the activity tracker with the Fitbit app to update the final daily steps data, and collected the mobile phone and charger. Participants were allowed to keep the tracker and encouraged to continue to maintain a daily exercise routine. Upon returning to the office, the RA logged in using the participant’s study Fitbit account credentials and downloaded the Fitbit steps data to the study computer.

### Acceptability and Feasibility Measures

Acceptability and feasibility were examined via tracking of implementation processes evaluation indicators, debriefing interviews, and postintervention satisfaction surveys.

#### Implementation Processes

An electronic database (REDCap) was developed to track usability issues [33]. This system contained data from multiple sources, including phone calls from participants, issues reported by the health coach, daily review of the mobile app back-end database, RA and project director tracking forms and notes, and timing of software updates for the activity tracker. Mobile app data were sent to the study’s secure database via encrypted transmission. If the mobile phone or app lost connectivity, data were transmitted the subsequent time the app was connected to the internet.

#### Coaching Call Indicators

The health coach recorded attendance and duration for the 5 calls. At every call, women were asked how many times in the past week they had synced their activity tracker with the trackC app and checked the app’s average daily steps graph and if they had experienced any problems doing this. On calls 1 and 3, women were asked how difficult they found it to use the graph (5-point response scale was 0=not at all to 5=difficult). After every session, the health coach was asked to rate how much of the material she felt the participant had understood (5-point response scale was 0=none to 5=all).

#### Debriefing Interviews

Semistructured debriefing interviews were conducted with a subset of participants to ask about their study experiences and suggestions for improvement. Selection of women was stratified to include those who had an iPhone versus other type of mobile phone or none, aged <50 versus ≥50 years, and from the 2 study sites. A trained bilingual-bicultural Latina interviewer (not the RA who conducted home visits) used an interview schedule that asked about their experience using the app (eg, what they did and did not like), ease of use, perceived usefulness for managing their cancer, and facilitating factors.

#### Satisfaction Survey

A 5-min satisfaction survey was administered at the final home visit after downloading participants’ activity tracker data for the study period and the final assessment. The survey asked them to rate the program’s perceived quality, ease of use, and usefulness. Overall quality of the app was assessed using a 5-level response set of “poor,” “fair,” “good,” “very good,” or “excellent.” Ease of use was assessed by asking about the overall difficulty of using the trackC app, syncing, and using the treatment summary, with response options of “not at all hard,” “a little hard,” “somewhat hard,” “quite hard,” or “very hard.” Perceived usefulness was assessed by asking participants to rate how useful the app and health coach were for helping them gain a sense of control over their health and how useful the app was for keeping their cancer treatment information in one place and knowing about cancer symptoms and treatment side effects to monitor. Response options for the usefulness ratings were “not at all,” “a little useful,” “somewhat useful,” “quite useful,” or “very useful.”

### Efficacy of Intervention Measures

To assess preliminary efficacy, we conducted baseline and 2-month interviews using structured surveys to examine changes in symptoms, knowledge, and well-being. Changes in pre- and postintervention average daily steps count were assessed based on activity tracker data.

#### Primary Outcomes

We measured 6 self-reported primary outcomes: 2 on symptoms, 3 on knowledge of cancer survivorship care, and 1 on self-efficacy for managing their cancer follow-up health care and self-care.

The 2 symptoms assessed were cancer-related fatigue and health distress. We adapted the *Patient-Reported Outcomes Measurement Information System* (PROMIS) Cancer-Fatigue Scale, which assesses the extent of fatigue and its impact on daily life over the past 7 days [34]. We dropped 1 item (“enough energy to exercise strenuously”) and added 2 items from the PROMIS Cancer Fatigue Short Form [35]: “felt tired when hadn’t done anything” and “limited social activities because of fatigue.” The final 7 items assess 4 aspects of severity (frequency that they felt tired, tired even when hadn’t done anything, extreme exhaustion, run out of energy) and 3 aspects of interference with daily life (frequency with which fatigue limited work, thinking clearly, taking bath or shower). To assess health distress, we selected 4 items from the Medical Outcomes Study Health Distress Scale [36] that asked how much of the time during the past month they felt discouraged, fearful, worried, or frustrated by their health problems. Response options for both fatigue and distress scales were as follows: “never,” “rarely,” “sometimes,” “often,” or “always.” Scale score were the mean of nonmissing items, with higher scores indicating greater fatigue effects (Cronbach alpha=.85) or health distress (Cronbach alpha=.91).

The 3 knowledge measures consisted of 2 global single item measures and 1 6-item scale. The 2 single items of global knowledge of survivorship care asked how true the following statements were for them: “you know what to expect now that your initial treatment has finished” and “you know how to take care of yourself after cancer.” The new scale assessed knowledge of follow-up care and ease of finding information. A sample item is “How true is the following statement for you: you know the possible side effects of your cancer treatment?” Response options for the 3 knowledge measures were 0=not at all true to 4=completely true. The scale was scored as the mean of nonmissing items with higher scores indicating greater knowledge (Cronbach alpha=.82).

A new 8-item self-efficacy for managing cancer care scale assessed confidence in ability to do what is needed to manage health care and health after cancer. A sample item is “How confident are you that you will be able to call your doctor if you have a question about a symptom that might be related to your cancer or treatment?” with response options of 0=not at all confident to 4=completely confident. The scale was scored as the mean of nonmissing items with higher scores indicating greater confidence (Cronbach alpha=.90). These new measures assessing women’s sense of control over their survivorship care drew on published questionnaires [37,38].

#### Secondary Outcomes

Secondary outcomes included emotional well-being, depressive and somatic symptoms, and average daily steps as recorded by the activity tracker.

Emotional well-being was assessed with the 6-item Emotional Well-Being Scale from the Functional Assessment of Cancer Treatment-General [39]. Scores range from 0 to 24, with higher scores indicating more well-being (Cronbach alpha=.77). We used the Patient Health Questionnaire 8-item version to assess depressive symptoms [40]. Scores range from 0 to 24, with higher scores indicating more depressive symptoms (Cronbach alpha=.64). We used the 6-item Brief Symptom Inventory Somatization Scale, which assesses the extent to which they were bothered by symptoms such as faintness and dizziness, pains in heart or chest, nausea, trouble getting their breath, numbness or tingling, and feeling weak [41]. Scores range from 0 to 4, with higher scores indicating more symptoms (Cronbach alpha=.76).

Baseline steps were calculated as the average daily steps during the 1-week run-in period (total steps divided by number of days) before the intervention start date. Postintervention steps were calculated as the average daily steps during the last week of the 2-month study period. Pre-post changes in average daily steps were calculated as the postintervention average daily steps minus the preintervention average daily steps.

### Analyses

Descriptive statistics were used to analyze sample characteristics and satisfaction survey responses. Debriefing interviews were transcribed verbatim in Spanish. A total of 3 bilingual-bicultural RAs independently performed content analyses of all transcripts, and discrepancies were resolved through team meetings. Linear mixed models were used to assess mean pre-post differences on primary and secondary outcomes; controlling for hospital site; and reporting unstandardized betas, *P* values, and Cohen *d* as an estimate of effect size.

## Results

### Demographic Characteristics

Of 100 women in the sampling frame, 23 enrolled in the study, 17 were ineligible, 17 could not be reached, 7 had incorrect contact information, 34 refused, and 2 were deceased. Mean age of participants was 55.8 years (SD 13.1), all were foreign-born and limited English proficient, most had an elementary school education or less (n=13), over half were of Mexican origin (n=16), and all had public health insurance (Table 1). About half (n=11) reported financial hardship in the past year, and most reported a comorbid chronic condition (n=17). The majority had breast conserving surgery (n=14) and both radiation and chemotherapy (n=15). Only 1 woman reported not owning a mobile phone.

**Table 1:** Descriptive characteristics of Spanish-speaking Latina breast cancer survivors participating in the Nuevo Amanecer (New Dawn) Survivorship Care Planning Program, Northern California (N=23).

Characteristics		Value
Age (years), mean (SD)		55.8 (13.1)
Years living in the United States, mean (SD)		20.1 (10)
**Educational attainment, n (%)**		
	Elementary or less or did not attend school	13 (57)
	More than elementary to high school graduate	5 (22)
	Some college or college graduate	5 (22)
**National origin, n (%)**		
	Mexican	16 (70)
	Central American	6 (26)
	South American	1 (4)
Married or living with a partner, n (%)		15 (65)
Employed full or part-time, n (%)		9 (39)
**Any financial hardship in past year, n (%)**		
	Yes	11 (48)
	No	11 (48)
	Missing	1 (4)
**Type of health insurance, n (%)**		
	MediCal (Medicaid in California)	19 (83)
	MediCal and Medicare	4 (17)
Presence of comorbid chronic condition, n (%)		17 (74)
**Time since diagnosis, n (%)**		
	1 year	8 (35)
	2 years	3 (13)
	3 years	5 (22)
	4-5 years	7 (31)
**Type of breast cancer, n (%)**		
	Invasive-ductal	15 (65)
	Ductal carcinoma in situ	3 (13)
	Other	5 (22)
**Surgery, n (%)**		
	Breast conserving surgery	14 (61)
	Mastectomy	8 (35)
	None	1 (4)
**Treatment, n (%)**		
	Adjuvant chemotherapy and radiation	9 (39)
	Neo-adjuvant chemotherapy and radiation	6 (26)
	Adjuvant chemotherapy only	1 (4)
	Adjuvant radiation only	3 (13)
	No treatment (no radiation nor chemotherapy)	4 (17)
**Self-rated health, n (%)**		
	Very good or excellent	14 (61)
	Poor or fair or good	9 (39)
Own a mobile phone, n (%)		22 (96)
Use mobile phone to make calls at least once a week during the last month, n (%)		22 (100)
Send a short message service text message using mobile phone at least once a week during last month, n (%)		14 (64)
Use mobile phone to access the internet, n (%)		15 (68)

### Acceptability and Feasibility

#### Implementation Processes

Nonscheduled home visits by the RA to all participants became necessary because participants requested help with the trackC app, activity tracker, or phone, or study staff noticed a lack of data transmission from trackC to the app backend database. A total of 63 nonscheduled visits occurred (mean=3 per participant, SD 1.9; range 1-7), during which the RA would troubleshoot technical and user issues and provide additional support and instruction. Most issues were related to technical (46 instances because of the app host site expiring, activity tracker software updates, or the app and tracker not syncing) or hardware-related problems (22 instances of activity tracker needing a new battery or the iPhone locking them out). Some were related to user issues (28 instances of forgetting to sync or how to do it, not knowing how to swipe out of an app section, or losing the activity tracker).

#### Coaching Call Indicators

Coaching calls lasted on average 15 min each (SD 3.4). A total of 19 of 23 participants (83%) completed all 5 coaching calls, 1 woman completed 4 calls, 1 woman completed 1 call, and 2 women completed no calls. Number of times per week that women synced their activity tracker and app ranged from 4.4 to 5.7. Number of times per week that women checked their daily steps graph on the app ranged from 4.2 to 5.9. Ratings of the difficulty with using the daily steps graphs at call 1 and call 3 were almost identical, with most women (12 at call 1, 11 at call 2) rating it as not at all difficult. A total of 3 women reported vision problems interfered with reading the app screens. On the basis of the coach’s ratings, the number of women understanding all of the material ranged from 17 (81%) for call 1 (daily steps and goal-setting) to 20 (100%) for call 3 (signs of recurrence).

#### Debriefing Interviews

A total of 10 semistructured postintervention debriefing interviews were conducted (Table 2). Participants were aged 56 years on average, and most were from Mexico (Mexico=7, Guatemala=2, and Nicaragua=1). All participants reported elementary school completion or less. In general, participants reported positive attitudes toward the program and increased awareness of the importance of walking. Themes emerging from the interviews are described next.

#### Perceived Usefulness of Intervention Components

Participants voiced appreciation for the trackC app information about their disease, treatments, side effects, and signs of recurrence, having felt misinformed about cancer survivorship before the study. All the women wanted the written SCP in addition to the app version. They reported feeling motivated and supported by the weekly check-ins with the health coach because she provided them with tailored, detailed, and credible information and support; helping them understand their disease, symptoms, and bodies; and achieve their walking goal. Participants valued the visual and auditory instant feedback provided by the activity tracker and app, for example, applause received after achieving their daily goal, helping them maintain a positive attitude toward walking.

#### Perceived Ease of Use of Mobile App

Participants described varied experiences about the effort required to navigate and use the app. Users with mobile phone experience found the app easy to use. However, 4 of 10 participants with little or no mobile phone experience expressed that use of the app required more effort and support at the beginning of the study. Some participants reported difficulties because of poor literacy or poor eyesight. All women reported being satisfied with the app’s interface, fonts, colors, and visuals.

#### Perceived Benefits of Intervention

Informants reported positive outcomes related to walking. A total of 7 of 10 women reported enhanced physical health because of their participation in the study, including weight loss, improved digestion and bowel movements, and improved sleep. Participants also reported improved emotional well-being, that is, decreased stress and better mood.

#### Social Norms

A total of 3 women felt a sense of accountability because they knew their steps were being monitored by themselves and others. Women reported that social support and encouragement from family members and neighbors pushed them to achieve their daily goal. Finally, several women expressed a shift from being extrinsically motivated by the app and coach to increase their walking to being intrinsically motivated because they wanted to do it for themselves.

**Table 2:** Themes from semistructured postintervention debriefing interviews of Spanish-speaking Latina breast cancer survivors participating in the Nuevo Amanecer (New Dawn) Survivorship Care Planning Program, Northern California (N=10).

Theme and subtheme		Illustrative quote	
**Perceived usefulness of intervention components**			
	App provided credible information about healthy lifestyles, side effects of treatments, and signs of recurrence.		“The app where you could find information you could trust. You see so many things on the internet, a home remedy, but nothing where you feel sure that what they are telling you is true.” (ID 9015)
	Feedback provided by activity tracker and app graph of daily steps progress over time were motivating		“What motivated me to walk was wearing the pedometer to see how much I could walk in one day and that this was recorded (on the app) so that I would not forget how much I had walked the day before and the day before that.” (ID 8027)
	Visual and auditory positive feedback from the app for steps taken (graphs of progress toward goal, cheering sounds) were motivating		“It seemed really important to me that when you met your goal, it was as if it (the applause) were saying, ‘Yay, you won!’ as if you had won a prize…and I liked it.” (ID 9015)
	Health coach provided detailed, tailored information on their specific treatments and potential side effects and follow-up care and motivation and support for walking		“Yes, she (health coach) really helps you. She motivates you to walk, how to take care of yourself, your health, what you should discuss with your doctor in case you feel something. She (health coach) tells you, you need to be aware of your body and report anything unusual, like pain, to the doctor. She gives you great advice.” (ID 8010)
	Goal setting provided motivation for walking		“Setting goals helped me focus...That helped me a lot. I used to not take my dogs for a walk, I would let them just run around here, but now I take my dogs for a walk so I can get more steps.” (ID 9001)
**Perceived ease of use of the mobile app**			
	Ease of use varied with prior experience using mobile phone		“It was a little hard, but then I read the instructions that they had given me. I have a cell phone, but I only use it for emergencies and to communicate with my children. But my cell phone is very basic and the one I use here (for the study) is more advanced. But after a while, I got the hang of it.” (ID 9002)
	Appearance, fonts, font size and colors—were satisfactory but a few suggested larger font and navigation buttons		“The button was in the corner and I would push it two or three times to get it to work. You need to have more room to be able to push the button.“ (ID 8040)
**Perceived benefits of the intervention**			
	More energy or less fatigue		“The walking is so good. I used to feel stressed, very tired, with no energy, and it all went away. At first, when I started walking, I would get tired, but now, I can’t believe it. After walking so much, I don’t get tired.” (ID 8010)
	Improved emotional well-being (less stressed or more relaxed, distracted from her illness, better mood)		“For me, a lot changed an awful lot. Pushing myself to walk a little more, I *saw* the difference in how much better, physically and emotionally. It’s a different type of relaxation. You get home tired and you say, “I am going to go for a walk! But, then you start, and it relaxes you *so much.* ” (ID 9015)
	Improved physical well-being (less pain, less constipation, lower blood pressure, weight loss, less leg swelling)		“Soon after I finished treatment, my legs would hurt a lot and get swollen. Once I started walking, it stopped. The pain and swelling went away. I weighed more and my legs hurt a lot and now my legs don’t hurt, I feel more motivated, and I have lost weight, and helped me feel less constipated.” (ID 9021)
	Improved sleep quality and quantity		“Now that I walk more, I feel really good, relaxed, I even sleep. I used to spend the entire night; it would be 2 or 3 in the morning, tossing and turning I could not sleep. And now, (laughs) I feel that the more I walk, the more I relax, I get tired and I know that I sleep, whereas before, I never slept.” (ID 9016)
	Improved body image		“I gained weight because of my cancer and treatment. At first it was hard for me to walk, but now I walk to work at least 3 days a week. Walking helped me feel better about my body. I even think I lost a little weight and I recently joined a gym.” (ID 9001)
	Increased self-efficacy		“I liked it all because I can sync and see how much I walked, primarily the effort I make to walk, and to see what I am capable of, that is, to keep making progress…My motivation became to see the level of effort I had made throughout the day to walk to improve my own health. I would make the effort and sometimes I would not reach the goal I wanted to reach and I would not like that, but then I would think, ‘tomorrow I have to do it.” (ID 8027)
**Social norms**			
	Shift in extrinsic to intrinsic motivation		“I still continue to walk. I have it in my head now always, as if they left me with a goal. I know that I reached the goal, but that is what I feel now. That the reason I walk is for my health and now I eat healthy food. Just yesterday, I walked over 17,500 steps.” (ID 9016)
	Walking was a commitment that they made upon joining the study		“Yes, I liked it because I felt as if I had a commitment to walk that I had to deliver on.” (ID 8040)
	Encouragement of family and friends		“I would get excited when I would open the app and the stars would come out. And my little boy would say, ‘Well, let’s go walk so we can see you meet your goal.’ And I would say, Yes, let’s go! And my kids would say, ‘Mami, aren’t you going to walk today?’” and I would answer, ‘Yes, go get me the cell phone’ (laughs). They, too, were involved.” (ID 9002)

### Satisfaction Survey

A total of 21 of 23 women completed the final assessment, for a retention rate of 91%.

#### Overall Quality

The majority of the women (17/21; 81%) rated the overall quality of the app as very good or excellent (all rated it as at least “good”). The overall quality of the information received on how to use the trackC app was rated as very good or excellent by 16 women (76%); all rated it as at least good.

#### Ease of Use

Most women (15/21; 71%) rated the ease of syncing the trackC app and activity tracker as being not at all hard (Table 3). Fewer respondents reported it being not at all hard to use the treatment summary found in the trackC app (11/21; 52%).

#### Usefulness

Regarding their ratings of the usefulness of the SCPP for feeling more in control of their health, all except for 1 woman rated the health coaching calls as quite or very useful, and all women reported the trackC app as quite or very useful. Almost all women (n=19) reported that the trackC app was quite or very useful for keeping their cancer treatment information in one place. Having information on trackC about cancer symptoms and side effects were both reported as being quite or very useful by 18 and 19 respondents.

### Efficacy of Intervention

#### Primary Outcomes

Regarding primary outcomes, compared with baseline, fatigue (B=–.26; *P*=.02; Cohen *d*=0.4) and health distress levels (B=–.36; *P*=.01; Cohen *d*=0.3) were significantly lower post intervention (Table 4). Women reported significantly greater knowledge of recommended follow-up care and resources after the intervention (B=.41; *P*=.03; Cohen *d*=0.5); self-efficacy for managing cancer follow-up care did not change.

#### Secondary Outcomes

Of the secondary outcomes, emotional well-being improved significantly post intervention (B=1.42; *P*=.02; Cohen *d*=0.3). Women’s average daily steps increased significantly from 6157 to 7469 (B=1311.8; *P*=.02; Cohen *d*=0.5).

**Table 3:** Satisfaction survey of participants completing the Nuevo Amanecer (New Dawn) Survivorship Care Planning Program, Northern California (n=21).

Domain				Value, n (%)
**Program evaluation: quality**				
	**How would you rate the overall quality of the trackC app?**			
		Excellent	7 (33)	
		Very good	10 (48)	
		Good	4 (19)	
	**How would you rate the overall quality of the information you received on how to use the trackC app?**			
		Excellent	4 (19)	
		Very good	12 (57)	
		Good	5 (24)	
**Program evaluation: ease of use**				
	**How hard was it to sync the trackC app and Fitbit?**			
		Not at all hard	15 (71)	
		A little hard	6 (29)	
	**How hard was it for you to use the treatment summary found in the trackC app?**			
		Not at all hard	11 (52)	
		A little hard	6 (29)	
		Somewhat hard	2 (10)	
		Very hard	1 (5)	
		Missing	1 (5)	
**Program evaluation: usefulness**				
	**How useful were the phone calls you had with the health coach for helping you feel as if you had more control over your health?**			
		Somewhat useful	1 (5)	
		Quite useful	6 (29)	
		Very useful	14 (67)	
	**How useful was the trackC app for helping you feel as if you had more control over your health?**			
		Quite useful	9 (43)	
		Very useful	12 (57)	
	**How useful was the trackC app for keeping your cancer treatment information in one place?**			
		Somewhat useful	1 (5)	
		Quite useful	7 (33)	
		Very useful	12 (57)	
		Missing	1 (5)	
	**How useful was the trackC app for knowing what cancer symptoms to look out for?**			
		Somewhat useful	1 (5)	
		Quite useful	8 (38)	
		Very useful	10 (48)	
		Missing	2 (10)	
	**How useful was the trackC app for knowing the side effects of the cancer treatments?**			
		A little useful	2 (10)	
		Quite useful	9 (43)	
		Very useful	10 (48)	

**Table 4:** Linear mixed model of pre-post changes in health outcomes and average daily steps count, controlling for site, Nuevo Amanecer (New Dawn) Survivorship Care Planning Program, Northern California (n=23).

Outcome measure		Preintervention, mean (SE)^a^	Postintervention, mean (SE)^a^	Unstandardized beta	*P* value	Cohen *d*
**Primary outcomes**						
	Fatigue^b^	2.21 (0.17)	1.95 (0.13)	–.26	.02	0.4
	Health distress^c^	2.32 (0.22)	1.96 (0.20)	–.36	.01	0.3
	Know what to expect after initial treatment ends^d^	1.14 (0.26)	1.10 (0.31)	–.035	.92	0
	Know how to take care of yourself after cancer^e^	1.93 (0.26)	1.74 (0.26)	–.194	.53	0.2
	Know about needed follow-up care and resources^f^	2.04 (0.20)	2.45 (0.15)	.41	.03	0.5
	Self-efficacy for managing cancer follow-up health care and self-care^g^	2.818 (0.19)	2.817 (0.16)	–.001	.99	0
**Secondary outcomes**						
	Emotional well-being^h^	18.72 (0.89)	2.14 (0.83)	1.42	.02	0.3
	Depressive symptoms^i^	4.68 (0.78)	5.30 (1.03)	.62	.43	0.2
	Somatization^j^	0.71 (0.14)	.69 (0.14)	–.02	.83	0
	Average daily steps^k^	6157 (526)	7469 (619)	1311.8	.02	0.5

^a^Controlling for study site and using intent-to-treat analysis (includes 2 participants who did not complete the postintervention survey).

^b^Adapted 7-item *Patient-Reported Outcomes Measurement Information System* Cancer Fatigue Scale-Short Form; possible range=1-5, high score=more fatigue.

^c^5-item subset of the Medical Outcomes Study Health Distress Scale; response options of 1=none of the time to 5=all of the time; possible range=1-5, high score=more health distress.

^d^New single item “How true is the following statement for you: you know what to expect now that your initial treatment has finished?” with response options of 0=not at all true to 4=completely true.

^e^New single item “How true is the following statement for you: you know how to take care of yourself after cancer?” with response options of 0=not at all true to 4=completely true.

^f^New 6-item knowledge of follow-up care scale with response options of 0=not at all true to 4=completely true; possible range=0-4, high score=greater knowledge.

^g^New 8-item self-efficacy for managing cancer care scale with response options of 0=not at all confident to 4=completely confident; possible range=0-4, high score=more confident.

^h^Emotional Well-being Scale of the Functional Assessment of Cancer Therapy-General; possible range=0-24, high score=better emotional well-being.

^i^Patient Health Questionnaire 8-item Scale; possible range=0-24, high score=more depressive symptoms.

^j^Brief Symptom Inventory Somatization Scale; possible range 0-4, high score=more symptoms.

^k^Calculated as the average daily steps during 1-week run-in period before intervention start and last week of the 2-month study period.

## Discussion

### Principal Findings

This study sought to develop and test the preliminary acceptability, feasibility, and efficacy of a multicomponent breast cancer SCPP designed for Spanish-speaking breast cancer survivors. The intervention consisted of a bilingual individualized written SCP, a Spanish language survivorship information booklet, a mobile app called trackC with an integrated activity tracker, and health coaching calls. We found preliminary support for the program, with significant 2-month improvements in fatigue, health distress, and emotional well-being and increased knowledge of recommended follow-up care and average daily steps.

Women reported checking their daily steps graph about 5 times per week and the majority indicated the app was not difficult to use. The majority of women rated the quality of the app as “very good or excellent.” Participants were motivated by the visual and auditory instant feedback provided by the activity tracker and app. In qualitative debriefing interviews, most women indicated that the app and coaching were useful for giving them a sense of control over their health, that the app provided a useful place for storing cancer and treatment information in one place, and that the SCPP resulted in increased physical activity, weight loss, and improved digestion and sleep. These results are consistent with similar studies that have demonstrated preliminary satisfaction with or interest in mobile phone app-based survivorship information among Latina [42] or non-white cancer survivors [43].

### Lessons Learned

Although women were receptive to the SCPP overall, we learned a number of lessons. First, women preferred receiving both the mobile and written versions of their bilingual SCP, so a mobile app alone might not suffice. Further customization of SCPs to include breast cancer type-specific information, for example, hormone receptor status, would be helpful. We were able to provide this level of customization via the health coaching, but this level of customization of the app exceeded the budget of this pilot study but could be addressed in future studies. We did not anticipate the extent of technical issues involved in maintaining communication between the trackC app, the activity tracker API, and the database management API. Unanticipated updates in the APIs of the activity tracker or database management system necessitated unscheduled home visits to install these updates as participants often did not know how to do this. Women sometimes forgot to wear the activity tracker or sync their trackC app and tracker. A small number of women with limited mobile phone experience, low literacy, or vision impairments indicated some difficulty in navigating the app, thus, the app would need to be further tailored and tested to meet their needs. For some women with limited iPhone or mobile phone experience, individualized assistance in learning how to use apps was needed; for example, knowing how to swipe to advance to next screen required repeated reinforcement. Regarding the design of the app, in the future, we would enlarge and centrally position the button used to sync the app and activity tracker app as suggested by some women.

### Limitations

This study has limitations. As a feasibility study, we did not include a control group and the sample size was small. As the study was conducted in Northern California with mostly Mexican women, results may not generalize to other regions or Latino national origin groups. In addition, because this was a multicomponent intervention, we are not able to isolate the relative effects of each of the components. Finally, we experienced a high refusal rate (60%), much higher than in our prior studies with women from the same population, so the final sample may be not be representative of Spanish-speaking Latinas in our region. Notably, this study coincided with a period of increasing immigration raids and heightened fear in local Latino communities. In our study, one of the most common reasons women gave for refusing to participate was fear that they would be tracked by immigration officials via the Fitbit wearable device.

Implications

Mobile phones offer promise as an excellent delivery mode among Latinos because of their widespread use of web-enabled phones to access the internet [25,44,45]. Mobile app interventions can be adapted for those with visual or auditory impairments and low literacy. Supplemental training and telephone health coaching can be provided to those with limited experience using mobile phones and to sustain levels of mobile app use. For many vulnerable populations, mHealth approaches alone may not suffice and more personal and intensive delivery modes will be needed. Some segments will prefer not to use mobile apps.

### Conclusions

Our pilot study results support investment in testing of smart phone and health coaching SCCPs among Spanish-speaking Latina breast cancer survivors. Additional research employing user-centered testing can identify the appropriate combinations of delivery modes and intensity of SCPPs for vulnerable subgroups of cancer survivors. Harnessing technology to address the needs of these groups ensures equitable access to its potential health benefits related to self-care and long-term cancer survivorship outcomes.

## Abbreviations

API: application programming interface
ASCO: American Clinical Society of Oncology
mHealth: mobile health
PROMIS: Patient-Reported Outcomes Measurement Information System
RA: research associate
SCP: survivorship care plan
SCPP: survivorship care planning program
